# Acute Myeloid Leukemia Presenting with Pulmonary Tuberculosis

**DOI:** 10.1155/2014/865909

**Published:** 2014-05-28

**Authors:** Merlin Thomas, Mushtak AlGherbawe

**Affiliations:** ^1^Pulmonary Medicine, Hamad General Hospital, P.O. Box 3050, Doha, Qatar; ^2^Department of Medicine, Hamad General Hospital, Doha, Qatar

## Abstract

We report the case of a 58-year-old immunocompetent man presenting with fever, cough, anorexia, weight loss, and cervical lymphadenopathy. Blood investigations revealed severe neutropenia with monocytosis. Chest imaging showed bilateral reticular infiltrates with mediastinal widening. Bronchoalveolar lavage culture and molecular test were positive for *Mycobacterium tuberculosis* and treatment with isoniazid, rifampicin, pyrazinamide, and ethambutol was started. Although pulmonary tuberculosis could explain this clinical presentation we suspected associated blood dyscrasias in view of significant monocytosis and mild splenomegaly. Bone marrow aspiration revealed acute myeloid leukemia. Thereafter the patient received induction chemotherapy and continued antituberculous treatment. After first induction of chemotherapy patient was in remission and successfully completed 6 months antituberculosis therapy without any complications. To our knowledge there has been no such case reported from the State of Qatar to date.

## 1. Introduction

Tuberculous infections are serious and life threatening conditions in patients with hematological malignancies [1]. There are several reports of tuberculosis occurring during treatment of hematological malignancies including leukemia, so high index of suspicion should be maintained in patients coming from endemic areas with clinical and radiological manifestations compatible with tuberculosis [2]. A delay in diagnosis of both could result in rapid deterioration and lethal outcome.

## 2. Case Presentation

A 58-year-old Filipino man presented with fever and cough for fifteen days. The symptoms began with sore throat followed by high grade intermittent fever and mucoid productive cough that was associated with anorexia, unquantified weight loss and fatigue. He did not consume alcohol nor had any illicit sexual relationships, and there was no history of travel or skin rash. He had no history of contact with tuberculous patient. On examination he looked pale, fully conscious, febrile with temperature of 38.9 centigrade, with no neck stiffness, and had bilateral firm matted supraclavicular lymph nodes with palpable spleen.

**Figure 1:**
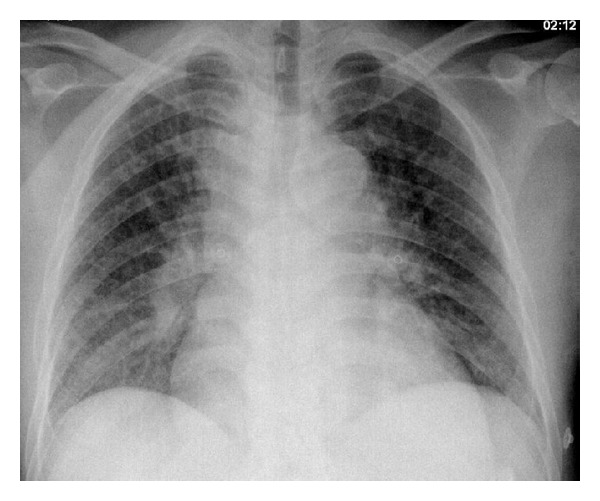
Roentgenogram of chest revealing bilateral reticular infiltrates with mediastinal widening suggestive of enlarged hilar and mediastinal lymph nodes. Enlarged cardiac shadow.

Blood counts revealed white blood cells 1900/microlitre and differential count neutropenia 200 microlitre with monocytosis (47%) reticulocyte (2.3%), macrocytic anemia with hemoglobin 6.1 g/L, mean corpuscular volume 120 fl, and normal thrombocyte count (Table 1). Blood culture, viral respiratory panel, and HIV serology were negative. PPD test was 16 mm. Chest X-ray (Figure 1) revealed mediastinal enlargement and bilateral reticular infiltrates. 3 consecutive sputum smears for acid-fast bacilli were negative. Bronchoscopy was done and bronchoalveolar lavage culture grew only mycobacterium tuberculosis and its genetic identity was confirmed by polymerase chain reaction (PCR) using gene expert targeting rpoB genes with wild type sequence. Endobronchial biopsy revealed necrotizing granulomatous inflammation* Mycobacterium TB* complex was sensitive to all four first line drugs.

**Figure 2:**
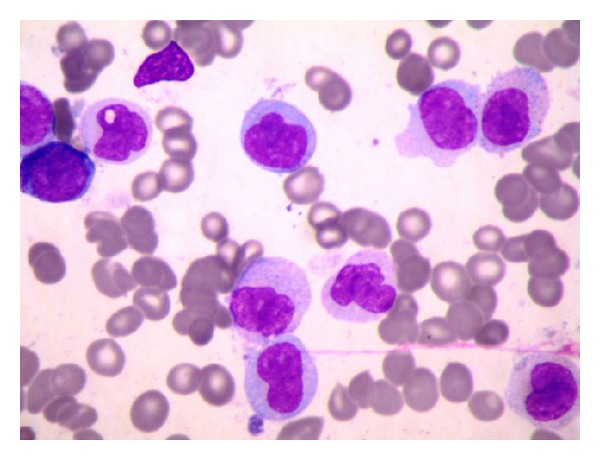
Severe macrocytic anemia with prominent rouleaux and dimorphism. WBC: severe neutropenia with absolute monocytosis. Fair number of immature cells.

**Figure 3:**
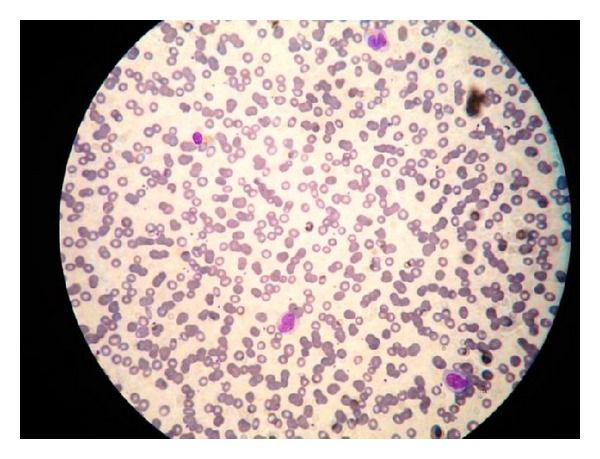
Bone marrow aspirate: Acute myeloid leukaemia with monocytic differentiation. The aspirate revealed leucopoiesis with prominent immature cells mainly monoblast and promonocytes occasional plasma cells. Depressed erythropoiesis with dyserythropoietic features.

Anemia workup revealed high ferritin 3048 mcg/L with normal vitamin B12 (325 pmol/L), homocysteine 10.5 micromol/L. Peripheral smear (Figure 2) revealed macrocytic anemia with neutropenia and rare immature cells. Hence bone marrow biopsy (Figure 3) was done to rule out any blood dyscrasia that revealed acute myeloid leukemia with 43% monoblast and promonocytes.

**Figure 4:**
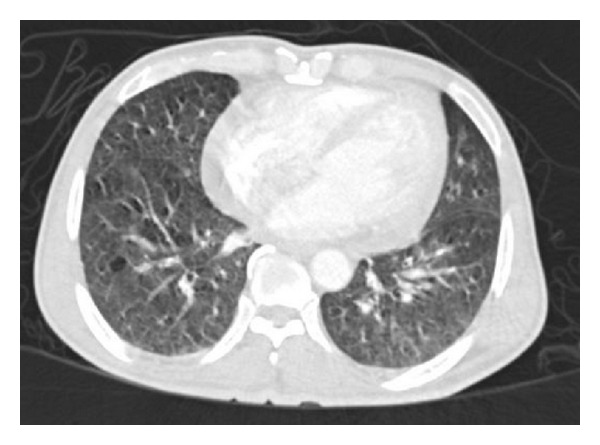
Computed tomography of chest: multiple nodular opacities in the upper lobe of right lung with interlobular interstitial thickening. Minimal pericardial effusion.

**Figure 5:**
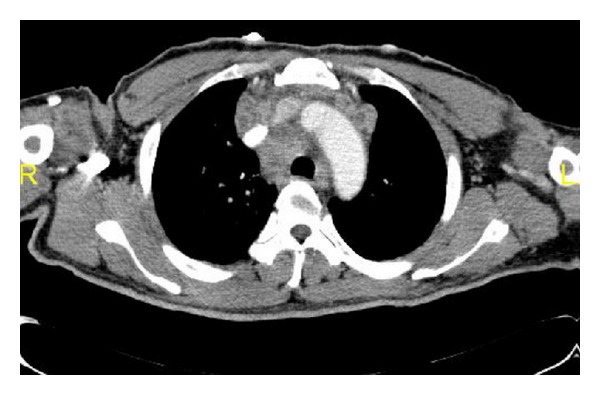
Computed tomography of chest showing mediastinal adenopathy with peripheral enhancement and central necrotic area.

Imaging by computed tomography (Figures 4 and 5) revealed marked mediastinal, hilar, and retroperitoneal lymphadenopathy. The lymph nodes showed peripheral enhancement with central necrotic area. Multiple nodular opacities in the upper lobe of right lung with interlobular interstitial thickening were observed. Multiple hypodensities in the spleen and minimal pericardial effusion were detected too.

Patient was managed in a protective environment and given meropenem for treatment of febrile neutropenia. Patient was started on four drug regimes for pulmonary tuberculosis that included isoniazid, rifampicin, pyrazinamide, and ethambutol with pyridoxine. Patient remained febrile throughout his hospital stay following which he was transferred to the oncology unit for chemotherapy. He received induction chemotherapy with cytarabine and idarubicin (3 + 7 protocol). Repeated bone marrow aspirate after first induction chemotherapy revealed less than 1% blast. Sputum gram stain and culture for tuberculosis at the end of 6 months of antituberculous treatment remains negative.

## 3. Discussion

Tuberculous infections (TIs) are serious and life-threatening complications in patients with malignant haematological disorders. It predominantly affects males with chronic myeloproliferative disorders, myelodysplasia, and AML [1]. Chen et al. and Mishra et al. reported that patients with AML rather than other types of hematologic malignancies had a higher rate of tuberculous infections due to* Mycobacterium tuberculosis* [2, 3]. It can precede or occur simultaneously or during treatment of hematological malignancies [4]; usually the prevalence in those patients ranges between 2.1 and 2.6% and when present it is usually disseminated [5].

The initial presentation in our patient was that of an infectious etiology. Tuberculosis was strongly considered in the differential diagnosis in view of his ethnicity and the presence of lymphadenopathy with reticular infiltrates in chest X ray. Anemia, leukocytosis, thrombocytosis, thrombocytopenia, and high erythrocyte sedimentation rate (ESR) are reported features in TB [6] and pancytopenia with bone marrow necrosis and granuloma formation is seen in miliary TB [7, 8]. In a study done in 95 patients with AML, tuberculosis as a cause of febrile neutropenia was identified only in 5.7% of the cases of AML [9], while in AML it is often associated with significant febrile neutropenia compared to non-AML patients [2] as in our patient. The human neutrophil peptides assist in the bactericidal action of the tuberculous bacilli and because neutrophils can mediate innate immunity against mycobacteria; the risk of TB was inversely associated with peripheral neutrophil counts in an adult tuberculosis cohort [10].

Most patients with haematological malignancies and pulmonary tuberculosis showed mediastinal lymphadenopathy, pleural effusions, and fibrocalcified lesions [2]. Andreu et al. reported that lymphadenopathy is the most characteristic radiological feature in tuberculosis and enhanced chest CT showing central hypodense area in hilar and mediastinal nodes support the diagnosis as seen in our patient [11]. Extrapulmonary presentation of TB disease is common in patients with haematological malignancies, ranging from 16% to 78% [4, 11, 12]. The presence of splenic hypodensities and pericardial effusions in our patient could be a manifestation of disseminated tuberculosis. Although the bone marrow biopsy was not giving the characteristic picture of caseating granulomas and no positive culture for TB was obtained from bone marrow, disseminated tuberculosis could be a coexistent feature in our patient.

Anti-TB drugs are associated with several adverse effects that include fever, leucopoenia, agranulocytosis, allergic reactions, and an elevation of liver enzymes [13, 14]. Fortunately, most of these toxic reactions are reversible [13]. Once anti-TB treatment is instituted, a successful outcome can be achieved in up to 90% of patients although a lethal outcome has been reported in patients with miliary TB [15]. There were no complications secondary to antituberculous treatment in our patients after the 6-month duration. Because TB is a serious infection in immunocompromised individuals, sometimes empirical anti-TB therapy is necessary when the clinical and the radiological features are strongly suggestive of TI particularly in patients living in endemic areas.

## 4. Conclusion

Tuberculosis by itself is a rare cause of febrile neutropenia and hence the associated presence of monocytosis and splenomegaly prompted us to proceed with bone marrow biopsy. A high index of suspicion should be maintained in immunocompromised individuals presenting with clinical and radiological manifestations compatible with TIs [16–18].

## Figures and Tables

**Table 1:** Blood test.

White cell count = 1.9 × 103/uL	Iron = 9 micromol/L
Neutophil = 0.2 × 103/uL	Total Iron binding capacity = 36 micromol/L
Monocyte = 0.01 × 103/uL	LDH = 460 U/L
Red cell count = 1.6 × 106/uL	Albumin = 34 g/L
Hemoglobin = 6.1 gm/dL MCV = 121 fl	Uric acid = 160 micromol/L
Platelet = 167 × 103/uL	ESR = 151 mm/hr
Blood urea = 4.8 mmol/L	CRP = 127 mg/L
Creatineane = 69 micromol/L	PT = 11.4 seconds
Total bilirubin = 8 micromol/L	APTT = 28.9 seconds
ALT = 31 U/L	Vitamin B12 = 328 pmol/L
AST = 51 U/L	
Alkaline phosphates = 137 U/L	
Total protein = 80 g/L	
